# The Principles and Practice of Modern Surgery

**Published:** 1907-11

**Authors:** 


### BOOKS AND AUTHORS.

**The Principles and Practice of Modern Surgery.** By **Roswell Park,** M.D., Professor of Surgery in the University of Buffalo, Buffalo, N. Y. In one imperial octavo volume of 1072 pages, with 722 engravings and 60 full-page plates in colors and monochrome. Lea Brothers & Co., Philadelphia and New York, 1907. (Cloth, $7.00, leather, $8.00, net prices.)

Here is a work which, by virtue of its intrinsic worth, takes rank as one of the better single volumes on surgery in print. Aside from its value as a scientific work, and in this respect it is the peer of many of the more pretentious works on the same subject, it has the added charm of the literary style of the author which strips from the more commonplace topics that dusty monotony which has become habit with most writers, and drapes them with a narrative elegance and interest which makes them all readable and most of them absolutely pleasing. It is this style, added to the high surgical attainments of the author and his ability to present the sum total of his vast experience in the clinic, which makes the work in its entirety a noteworthy book.

Before examining the book proper it will be well to carefully read the preface and introduction, wherein is given an insight into the author's methods. The preface says: "There is still room for many a serious effort to place the subject (surgery) before students and practitioners, in a way to instruct from the beginning through to the operative and post operative treatment. This has been the object of the present volume upon which the author has brought to bear the experience of many years as a teacher and surgeon, and into which he has also endeavored to infuse the most advanced knowledge gleaned from the surgical literature of America and Europe. To the extent of the author's ability the work, therefore, presents the net surgery of today, obsolete and obsolescent material having been excluded, and the pages being devoted to sound principles and practice, stated as clearly and succinctly as possible."

The introduction gleams with epigrams. "During the past 30 years surgery, thanks to eminent workers in the surgical laboratories of the world, has made progress scarcely equaled by the science of electricity, and the impossibilities of yesterday have become the routine of today." "The surgeon and the physician have drifted too far apart. It is time they met again in the presence of the pathologist."

Here, too, is indicated the character of the book, in the statement that preference is given to those operations which have best served the author in his personal experience; so there is no need to anticipate that this book is like some of the other efforts in surgical bookmaking, a literary rag-bag of might-bes and ought-to-bes, trimmed with vague and untried theory. Throughout the book there is a sustained effort, and let us pray that it may bear fruit as it deserves, to simplify and rearrange some of the more flagrant nuisances of surgical nomenclature. For instance, Dr. Park calls attention to the abuse of the term "lymph glands" which he very properly replaces with "lymph nodes." It is, of course, wholly impossible to review such a masterly work in its entirety in a journal not altogether devoted to surgery; but those portions which make it what it is, by all odds the better surgery of today, deserve explicit mention.

The present volume is in no sense a new edition of the surgery issued some years ago under the editorship of Dr. Park. It is an individual work, wholly the author's and represents his personal views. Neither is it an encyclopedic volume; hence those who may feel inclined to criticism because it is not a catch-all will feel the tender tooth of disappointment. In the earliest pages one is struck by the clearness with which the author distinguishes clinically between hyperemia and inflammation, the first instance so far as is known by the reviewer, wherein a textbook makes inflammation wholly and absolutely the result of infection. Pathologists may produce artificial inflammation by other means than bacteria; but, clinically viewed, inflammation is the result of bacterial invasion and nothing else.

An entire chapter is devoted to the staticus lymphaticus and this is the first definite textbook presentation of the subject which is considered in all its phases, and is given as the cause of many of the instances of sudden death after anesthesia, and during convalescence from various diseases. The condition is described in detail, its danger signals are pointed out and the way cleared for the avoidance of such distressing accidents as have happened in the past during comparatively insignificant operative procedures. The diagnosis is made clear and the treatment is outlined in so simple a manner that he would be negligent, to say the least, who closed his eyes to the condition.

There is a distinct advance in the chapters devoted to shock and collapse which, by the way, are entitled, Disturbances of Blood Pressure. Here are given the newer views of shock and collapse following out Crile's investigations of two years ago, and there has evidently been much serious work done in carrying forward his investigations. At a single sweep of bold statement, backed by liberal experience, the use of alcohol, digitalis and the rest of the hurry-up remedies of other days, is done away with and there is substituted the use of external heat, adrenalin and salt solution. In serious cases, where possible, the use of the Crile pneumatic suit is advocated. There is sounded a note of warning in connection with the modern treatment of shock and collapse concerning the indiscriminate intravenous use of salt solution. The question is one of greatest importance and if everyone who reads the book did no more than follow the teachings as laid down in these chapters, there would be performed a great service to humanity. Its conservative, sensible presentation of simple fact is convincing and cannot be too highly commended.

Possibly, when he reaches part five, some supersensitive critic may take exception to the caption "Cysts and tumors" on the ground that a cyst is a tumor, hence the designation is superfluous. Let him read these definitions: "A tumor is a new formation not of inflammatory origin characterised by more or less histological conformity to the tissue in which it has originated and having no physiological function." "A cyst is a tumor containing one or more cavities filled with fluid or semifluid contents." Beyond question this section contains the best classification of tumors and the best article on cysts and tumors which has yet appeared in any work on surgery. The author's views as regards the distinction between sarcomatous and carcinomatous growths are direct and convincing, and will entirely replace the views of Bland-

Sutton which heretofore have been considered classic. Dr. Park makes large use of the term "infectious granulomata" denoting new growths of a distinctive class of neoplasm of inflammatory origin.

The parasitic theory of carcinoma is upheld and, while Dr. Park does not by any means claim the parasite has been discovered or that such discovery is about to be made, he feels assured that the agent is probably protozoan in character. Of more than unusual interest are these statements which he makes in conclusion: that cancer is at first purely a local condition; that it is not transmitted by inheritance; and that every cancer is curable if recognised early and totally removed. As the result of years of research on the part of the author in this particular field, these statements cannot be passed over lightly; they must be accepted at their full worth as the opinions of a man qualified to speak with authority.

In the chapter devoted to "Deformities due to congenital defects or acquired disease of the locomotor apparatus," Dr. Park introduces an innovation into surgical teaching. Usually one finds the very important and too frequently slighted subject of orthopedics pushed aside or relegated to a few slipshod paragraphs at the end of a book. In this work the matter is given a prominent place and under the title given above is most ably discussed and exceptionally well illustrated. There is really more genuine information of an assimilable character in this chapter than one finds in most of the books devoted to the subject as a specialty, for it lacks the stilted, academic description and the monotony of devious detail.

It is safe to say in considering the chapters on fractures that nowhere else is the subject given such careful consideration in connection with fractures of the upper extremity, for which the author recommends the plaster of paris splint in every case to the absolute rejection of the ready made splints of various materials. He shows how the appliance can be made and describes its advantages over all other kinds by merely using, with intelligence, plaster of paris and surgeon's lint. The result is a splint which is of use for any fracture of the upper extremity of whatever character; a splint which is quickly made to order for each individual case and is valueless for any other. It makes a better and more satisfactory dressing than any yet devised for such injuries, and is a distinct surgical advance.

Park's operation for hernia is described in detail for the first time. The main feature of improvement over existing operations is that the author uses the twisted and divided sac as suture material for closing the ring. The various steps of the operation are freely illustrated and there is little doubt that it will become popular. Theoretically it might in some quarters be considered of secondary importance, this use of the sac; but the manifest advantages of making use of the patient's own tissue as suture material, viable, strong and unquestionably clean, will not be overlooked by the unbiased reader, whether he be practitioner or student. Rather startling is the author's acceptance of the Russell theory as to the etiology of hernia which, in brief, is that every hernia, no matter what its location, is of congenital origin, even if the tumor does not appear until late in life. Both Coley and Bull have recently shown a marked leaning toward the acceptance of this theory, but Dr. Park is the first American surgeon to accept it and give it standing in a textbook. If, as he says, a hernia means the presence of a sac and that a sac can appear only as the result of a congenital deficiency, then there is little left for argument as to the congenital origin of hernia.

In most surgery textbooks there has always been a noticeable deficiency in the handling of what is popularly known as "railroad spine." Growing more important every day owing to legal questions constantly arising, the injury, or belief, or however one may personally desire to classify it, deserves all the space which Dr. Park gives it and the importance which he attaches to it. The question is discussed logically in all its phases, and with none of the hysteric tendency which crops out in legal treatises.

To the term "ileus" which has been bandied about learnedly by writers for sometime past and variously defined as being this, that, and the other condition of abdominal origin, sometimes as a specifically located trouble at others as an illy defined pain of vague origin, Dr. Park has one definition and that is acute intestinal obstruction or strangulation. He very properly deplores the unsettled condition of the term and throughout his book uses it interchangeably with the condition described and no other. It is the best and most common sense view to take of the condition and its adoption will do much to eliminate from surgical writings the careless uses to which it has been put in the descriptive field of abdominal disorders.

Not the least important portion of the book is that devoted to the preparation of patients for operation and their after treatment. This is especially valuable for students and without too much of a strain on the pride of many a practitioner it may be absorbed and become of inestimable value. There is none of that heavy lidded descriptive writing here; it is all plain, frank. simple, and usable information. Really, this is something of an innovation in textbooks, for the majority of them dismiss the subject of preparation and after treatment in a few pleasing paragraphs. The ante-and post-operative treatment of a surgical patient are quite as important as the main surgical procedure, and it is not likely that the wormeaten joke about the operation being successful but the patient dying, would have such a vogue among cute and clever lay people, had more attention been paid to getting the patient ready for operation and giving him scientific care after he left the table. An entire chapter is devoted to each division of this subject in Dr. Park's book, and questions of vital importance to the patient are considered in detail. The suggestions and the advice given cover almost every possible contingency. Nothing seems to be too insignificant for discussion even to the dissipation of the excessive postoperative thirst which is frequently so distressing to a patient.

Those sections of the book which are devoted to genitourinary and venereal diseases are especially worthy of commendation, and it is rather more than merely gratifying to find a general surgeon pointing out with such minute care the diganosis of renal colic, instead of simply passing it over as a condition which ought to be recognised without much trouble. The diagnostic points mentioned are conservative and well defined. The mention of Kelly's suggestion to instil a sterile solution into the kidney pelvis for the purpose of diagnosis in suspected renal pain of obscure origin, will meet with approval in Baltimore beyond question, but it is hardly a satisfying procedure and belongs among the freaks.

In dealing with prostatectomy the author covers the entire question in limited space most completely and leaves nothing to be desired. What will strike one as especially graceful is the footnote regarding the credit for the suprapubic operation, which has so long and so erroneously been known as Freyer's operation. The note in question gives the credit to Fuller of New York. This matter was editorially threshed out in The Journal a year or two ago after a most painstaking investigation of an independent character, and the operation was placed to the credit of Fuller of New York, although Ramon Guiteras modified it somewhat at a later day, but before Freyer did his first operation which was a replica of the Fuller-Guiteras procedure.

The publishers deserve great credit for the manner in which the book has been placed before the profession. The binding is of superior workmanship, the spring back making it possible to open the pages at any place and have the book remain flat-paged. The illustrations, in the main, have been selected with apparent care and the technical workmanship cannot be criticised. Many of the plates are reproductions of cases from the Buffalo clinic. An intensely interesting series of plates are those supplied by Major Charles Lynch of the Army medical department, showing gunshot wounds received during the Japanese-Russian war and radiographs of shell and bullet wounds. Some of the most excellent illustrative work in the entire volume is the series of skiagrams made by Dr. Plummer, of Buffalo, showing among others some of the cases of foreign bodies in the esophagus and trachea from Dr. Park's clinic.

Being essentially a surgery the book does not go into any of the special departments such as gynecology, nose, throat, eye and ear diseases. Only those surgical affections which have to do with the external portions are considered, and this elimination of these specialties adds to the dignity of the book.

It would be surprising to search a book of the magnitude of this surgery and not find an error of some form, yet in all the pages there were found only two, and they are beyond question of typographic origin. One is where the "internal" operation in appendicitis is spoken of, where "interval" is clearly meant; the other is in the chapter on vesical calculi wherein it is stated that calculi escape from the bladder when they are small enough to pass through the "ureter," instead of "urethra".

If one will but go back to what the author says in his introduction he will not wonder at the absence of many things which have been occupying considerable space in the medical journals during the last year or two. The book is in no sense intended as an encyclopedic guide to surgical literature, operations or procedures. It is an individual work representing the best there is in modern surgery and presenting only those things which time and experience have proven to be of value. Hence, there will be found lacking from its pages any serious mention of Crile's transfusion, Murphy's nerve surgery or of the opsonic theory, the latter being dismissed with a mere note. So many of these, like many other procedures, are as yet in the experimental stage, and have not sufficiently crystallised to make them either safe or valuable for presentation to the profession in a textbook on modern surgery. Park's Modern Surgery, in a word, is a model, modern book.

